# Chemical, electrochemical, and quantum investigation into the use of an organophosphorus derivative to inhibit copper corrosion in acidic environments

**DOI:** 10.1038/s41598-024-60614-5

**Published:** 2024-05-18

**Authors:** M. A. Deyab, Jwaher M. AlGhamdi, Marwa M. Abdeen, Marwa Abd Elfattah, Ahmed Galhoum, Omnia A. A. El-Shamy, Ibrahim E. El-Sayed

**Affiliations:** 1 Egyptian Petroleum Research Institute, Nasr City, Cairo, 11727 Egypt; 2 Department of Chemistry, College of Science, Imam Abdulrahman Bin Faisal University, 31451 Dammam, Saudi Arabia; 3 Basic Science Department, Higher Institute of Engineering and Technology, Menoufia, Egypt; 4 Chemical Engineering Department, Higher Institute of Engineering and Technology, Menoufia, Egypt; 5 Nuclear Materials Authority, El-Maadi, P.O. Box 530, Cairo, Egypt; 6 Chemistry Department, Faculty of Science, Menoufia University, Shebin El-Kom, Egypt

**Keywords:** Organophosphorus derivative, Copper, Acid solutions, Corrosion, Quantum studies, Chemistry, Electrochemistry

## Abstract

In order to protect the copper against corrosion, a novel corrosion inhibitor known as diphenyl ((2-aminoethyl) amino) (4-methoxyphenyl) methyl) phosphonate (DAMP) was developed. Acid solutions of HCl and H_2_SO_4_ were the aggressive solutions employed in this study. Analysis using the FT-IR, ^1^H-NMR, ^31^P-NMR, ^13^C-NMR and BET confirmed that the DAMP was successfully synthesized. The anti-corrosion capabilities of DAMP are evaluated using a combination of chemical, electrochemical and quantum studies. The DAMP has been found to be crucial in preventing the corrosion of copper in both HCl and H_2_SO_4_ acid. This was obviously implied by the observation that the corrosion rate of copper in acid solutions decreased when DAMP was added. It is significant to note that 180 ppm produced the highest levels of inhibiting efficiency (96.6% for HCl and 95.2% for H_2_SO_4_). The tendency of DAMP to adsorb on the surface of copper through its hetero-atoms (O, N, and P) is the main factor for the anti-corrosion capabilities of DAMP. Results from SEM/EDX tests supported this. The actual adsorption takes place via various active centers, physical and chemical mechanisms that are coordinated with the estimated quantum parameters. Additionally, the adsorption of DAMP adheres to the Langmuir isotherm.

## Introduction

For its beneficial properties including conductivity, flexibility, and resistance, copper and its alloys are well known. This makes them appropriate for a diversity of applications in the production of wire, sheets, and pipelines for the electronic industry, marine industry, power plants, heat exchangers, and cooling towers^1,2^. Copper becomes a noble metal with sufficient corrosion resistance in surroundings as well as some chemical conditions when a protective passive (oxide) sheet or inert layer of corrosion products formed on its surface^3,4^. Depending on the environmental conditions, the pitting corrosion may be occurred on copper surface in the presence of oxygen and some aggressive anions such as chloride and sulfate ions^5–7^.

A system made of copper can perform poorly and lose efficiency as a result of copper corrosion and the production of corrosion products on its surface. Due to the widespread use of copper in various industries, numerous studies have been conducted and are continuously being done in the field of study on copper corrosion and corrosion prevention. There is a schematic depiction of various copper-based businesses that are subject to corrosion assaults^8^.

A common method for reducing or preventing the rate of corrosion of metallic substrate in acidic conditions is the use of corrosion inhibitors^9–13^. These are chemicals that, when added to corrosive medium in modest quantities reduce or stop the reaction of metal with the media. Organic or inorganic materials are also possible^14^. The organic counterpart has properties like hetero atoms and/or double bonds, a large area of surface, a charged center, etc. this, upon adsorption on the metal surface, will cover a significant portion of the metal with that facilitate film-forming on the metal surfaces and subsequently isolate it from the corrosive ions that are present in the environment^15–23^

It is generally known that a good corrosion inhibitor must work effectively even at small dosages. Due to their resistance to microbial destruction, low toxicity, action in watery environments, and stability against degradation, phosphonates are useful and fascinating inhibitors^24,25^. sp^3^ hybridized nitrogen atoms in these compounds boost the inhibitory effect^25^. Many phosphorus compounds, such as phosphonic acids^26^, aminomethylene phosphonates^27^, pyrazine derivatives^28^, and α-aminophosphonate^29^, as well as nitrogen-containing molecules, such as imidazolines, amides, and amidoamines^30^, are used as corrosion inhibitors. Organophosphorus compounds and derivatives, such as α-aminophosphonate (contains P, N, and O as hetero atoms), have been extensively studied for metal recovery, water purification, and metal removal owing to their excellent binding characteristics with metal ions.^31–34^.

The geometric and electrical molecular structure of a molecule affects how successful it is in a chemical process^35^. The ideal way in which the inhibitor might cover the metal surface is related to the geometry of the molecule, which subsequently has a significant impact on the adsorption of the inhibitor on the metal surface. The key quantum properties of the evaluated inhibitors are investigated using a variety of theoretical techniques. Density functional theory (DFT) is a quantum mechanical computation that is frequently used to evaluate experimental data and determine structural characteristics for even extremely complex molecules.

The first objective of this work is synthesis of a novel DAMP compound throw an easy, economical way and high yield. This compound is prepared using a one-pot synthesis procedure and direct reaction of ethylene diamine with anisaldehyde and triphenylphosphite. The structural and functional characteristics of DAMP is investigated by FT-IR, ^1^H-NMR,^13^C-NMR, ^31^P-NMR, and BET. The Second objective of this work is to evaluate the inhibitory action of DAMP against the corrosion of copper in 1 M sulfuric and hydrochloric acid solutions. During this study chemical and electrochemical methods were used. These methods consist of determining the corrosion rate and inhibition efficiency. The state of inhibitor adsorption is monitored by studying the concentration, immersion time and temperature effects. Characterization techniques, namely scanning electron microscopy (SEM) coupled to energy dispersive X-ray spectrometry (EDX), Fourier transform infrared (FT-IR) were performed to describe the morphology and the surface roughness of the examined copper samples when they are inhibited and uninhibited. Additionally, theoretical simulations made with DFT are used to show how the electronic structure of the inhibitor and its corrosion behavior are related.

According to studies on the toxicity impact of corrosion inhibitors, α-aminophosphonates have little toxicity to human cells due to their structural resemblance to natural α-amino acids^36,37.^

## Experimental

### Chemicals and materials

The following ingredients were supplied by Sigma-Aldrich (Saint-Louis, MS, USA): anisaldehyde, ethylenediamine, and triphenylphosphite. Lithium perchlorate and acetonitrile were purchased from Fluka AG in Buchs, Switzerland. Without any purification, all reagents were used just as they were delivered.

The 99.99% copper samples (size = 2 cm × 3 cm × 0.1 cm) and cylinder rod with circular cross section (area = 1.0 cm^2^) were used to calculate the corrosion rate in chemical and electrochemical tests, respectively. The samples were repeatedly polished with fine-grade emery paper, cleaned with acetone, washed with doubly-distilled water, and then dried.

Commercial hydrochloric and sulfuric acid solutions from Sigma Aldrich, each having a concentration of 34% and 96%, were employed as the acid solutions. The analytical HCl, H_2_SO_4_ stock was diluted with distilled water to create the test solutions with a 1.0 M concentration. Inhibitor stock solution was prepared from synthesized DAMP inhibitor. The DAMP inhibitor was dissolved in 1.0 M solutions of hydrochloric and sulfuric acids. The compound dissolves easily in the acid solutions without further using of organic solvents. As a result of the presence of an amine group, this allows the formation of a salt with the acid, which facilitates the dissolution process. The working solutions were prepared by appropriate dilution of the stock solution immediately prior to use.

### DAMP compound synthesis and characterization

The following were dissolved in CH_3_CN (5 ml): anisaldehyde (1 mmol), ethylenediamine (1 mmol), and triphenylphosphite (1 mmol). Before adding the Lewis acid catalyst (LiClO_4_, 20 mg), the mixture was agitated at room temperature for 10 min. The solution was stirred until the TLC confirmed that the reaction was finished. The desired diphenyl (((2-aminoethyl) amino) (4-methoxyphenyl) methyl) phosphonate (DAMP) was made by air-drying the final product after they had been filtered and collected with high yield (93%). The materials were purified by dissolving the as-prepared solid in chloroform or methanol and then recrystallizing them. Before use, the dry powders were lastly kept in a desiccator.

A JEOL ECA-500II spectrometer is used to measure ^1^H-NMR and ^13^C-NMR (solvent DMSO-d6) at 500 MHz and 100 MHz, respectively. Chemical alterations that is ppm-proportional to the solvent that is connected. A BRUKER spectrometer (Japan) is used to detect ^31^P-NMR spectra at 162 MHz in DMSO-d6. The surface area was calculated using N_2_-adsorption–desorption isotherms recorded on a Quanta Chrome Nova 3200 instrument (Boynton Beach, FL, USA) under a degassing temperature of 60 °C for 3 h. The pore volume was calculated using the BJH method.

### Weight loss and electrochemical measurements

Weight loss is the most popular and straightforward approach for determining how effectively corrosion is inhibited. Corrosion alters a variety of attributes, including mass, electrical resistance, magnetic flux, and mechanical qualities. The method of utilizing a weight loss coupon to assess corrosion damage is highly helpful for monitoring corrosion as well as for looking into environmental conditions that cannot be replicated in a lab. Undeniably, using this process to determine the corrosion inhibition efficacy is helpful. In this low-cost method, tiny samples are submerged in corrosive fluid for a specific period of time before being removed. The distinction between the mass before and after immersion is crucial. Copper samples were weighed and then submerged for 24 h in 100 ml of 1.0 M HCl and H_2_SO_4_ solutions with various doses of DAMP. Samples were taken at the end of the procedure, cleaned with distilled water, and dried. In this method, we averaged three trial measurements using a Mettler H35AR digital analytical balance. The following relation was used to determine the rate of corrosion (*C*_R_)^37,38^.

1 $$C_{{\text{R}}} = W/(A \times t)$$

where, *W*: the average weight loss and, *A*: total surface area of copper samples, and *t* is the period of the immersion.

The following equation was used to compute the DAMP inhibitor's inhibition efficiency (*E*_W_%) in the applied aggressive solutions:

2 $$E_{{\text{w}}} \% = \frac{{C_{{{\text{R}}0}} - C_{{\text{R}}} }}{{C_{{{\text{R}}0}} }} \times 100$$

where *C*_R0_ and *C*_R_: the corrosion rate of copper without and with DAMP inhibitor, respectively.

The electrochemical processes were carried out using a standard glass cell with three electrodes. A sizable platinum sheet served as the counter electrode and a saturated calomel electrode (SCE) served as the reference electrode. The open-circuit potential was collected for 30 min to reach a steady state potential prior to the electrochemical tests^39^. The potentiodynamic polarization (PP) scan was typically captured by varying the potential between + 0.250 and −0.250 V (vs. OCP) with scan rate 0.125 mV/s. Electrochemical impedance spectroscopy (EIS) tests were performed at an open circuit potential with a voltage amplitude of 10 mV and frequencies ranging from 0.01 to 100 kHz.

### Theoretical calculations

Using the software HyberChem 8.0.10, quantum computations of the under-investigation molecule were carried out in a vacuum. The investigated DAMP molecule is geometrically optimized (see Table 1) before starting the calculation and the total energy found to be (E_Total_ = −109,351 kcal/mol). All calculated parameters are obtained using DFT/B3LYP and basis set of 6-31G and applying the Unrestricted Hartree–Fock (UHF).

**Table 1:** Quantum descriptors for the DAMP molecule.

Optimized Structure	The obtained and calculated function
	Dipole Moment = 3.882 Debyes
	E_H_ = −8.519 eV
	E_L_ = −1.839 eV
	ΔE_g_ = E_H_–E_L_ = 6.680 eV
	η = −0.5(E_H_–E_L_) = 3.34 eV
	σ = 1/η = 0.299 eV^-1^
	ΔE_b-d_ = −η/4 = −0.835 eV

### Surface characterization

SEM–EDX microanalysis detectors (series: 1200 EX II electron microscope: JEOL-JEM) were used to determine the surface condition of a copper sample that had been submerged for 24 h in 1 M HCl and H_2_SO_4_ solutions without and with inhibitor. These investigations provided information on the surface shape and elemental makeup of the species that had developed on the metal surface. Using a Fourier Transform Infrared FT-IR spectrometer, we looked at the chemical make-up of the inhibitor and the sample surface when it was exposed to the inhibitor. FT-IR spectra are captured using a Thermo-Fisher Nicolet IS10 (Waltham, MA, USA) spectrometer in the 4000–400 cm^−1^ region.

## Results and discussions

### Synthesis and characterization of DAMP

#### Synthesis of new DAMP inhibitor

**Figure 1:**
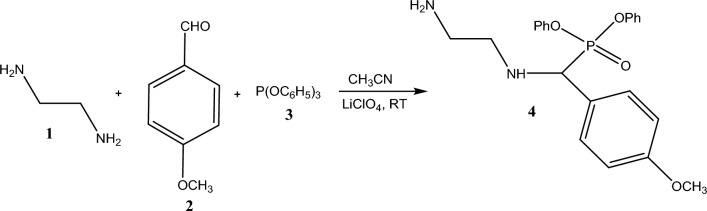
Synthesis route for DAMP preparation.

Figure 1 illustrates the synthesis route for the preparation of DAMP.

This simple one-port reaction occurs between amine compound (ethylene diamine) and both anislaldehyde and triphenylphosphite; LiClO_4_ is used as the catalyst for the conversion into inhibitor. Scheme S1 (see Additional Material Section) shows the suggested mechanism for the molecular interactions between the reagents for producing the DAMP inhibitor. The first step consists of the formation of an imine-intermediate, followed by the attack of this intermediate by nucleophilic phosphite (leading to the formation of phosphonium ion). This reaction is catalyzed by the presence of the Lewis acid catalyst (i.e., LiClO_4_). Herein, the reaction was carried out in polar aprotic solvent such as acetonitrile (CH_3_CN) with reactants bearing polar groups and non-polar ones and all dissolved well to form a homogenous solution. Once the product starts to form (after the addition of the catalyst) the hydrophilicity decreases; indeed, the synthesized product becomes progressively hydrophobic and begins to precipitate from the solvent phase. In the last step of the process, the reaction of phosphonium intermediates with water promotes the elimination of phenol and the formation of the relevant α-aminophosphonate with a yield 93%.

### ^1^H-NMR, ^31^P-NMR and ^13^C-NMR studies

Figure S1 shows ^1^H-NMR (DMSO-d6, 500 MHz): δ 2.59 (m, 2H, CH_2_), δ 2.76 (s, 2H, NH_2_), δ 2.91 (m, 2H, CH_2_), δ 3.7 (s, 3H, OCH_3_), δ 4.23 (m, 1H, CHP), δ 6.73–7.93 (m, 14H, Ar–H). δ 8.9 (br, 1H, NH).

The appearance of a singlet peak at = 22.02 ppm in the ^31^P-NMR spectrum (Fig. S2), which is present in the DMSO, 162.0 MHz, verifies the presence of the phosphonate moiety linked with the (P-O) signal.

Furthermore, the ^13^C-NMR (100 MHz, DMSO-d6) results in a number of carbon peaks that are compatible with the various surroundings of material's carbon atoms (Fig. S3). The loss of the (C = O) peak for anisaldehyde, which was measured at roughly 193.04 ppm, and the appearance of the (P–CH) Chiral carbon atom peak as a doublet at approximately 62.97 ppm serve as indicators of the alteration. This doublet exhibits strong carbon-phosphorus coupling. this doublet is characterized by a large coupling constant (Jpc = 127.1 HZ). The aliphatic chain appears at *δ* (ppm): 38.84 (CH_2_NH_2_), 49.5(CH_2_N–CHP), 55.64 (OCH_3_) while the aromatic carbons appear at 114.5, 115.84, 116.21, 118.33, 129.16, 135.83, 136.38, 136.8, 153.27, 153.82, 154.28 ppm.

### Textural analysis (BET) studies

**Figure 2:**
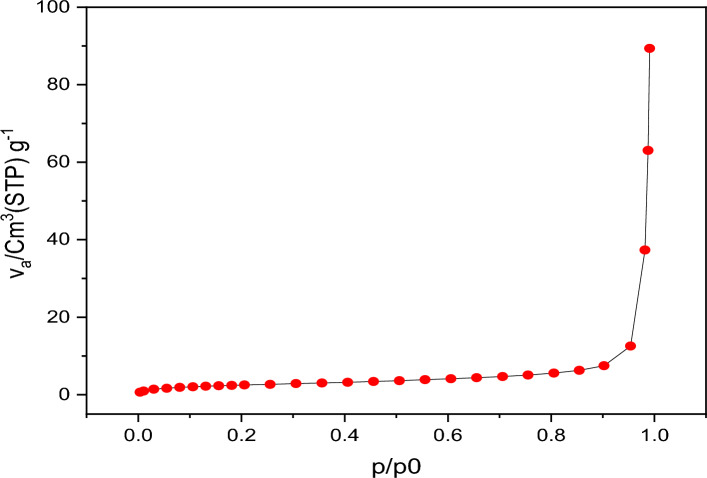
N_2_ adsorption/desorption isotherm for the DAMP inhibitor.

The nitrogen adsorption–desorption isotherms were used to calculate the accurate surface areas, pore volume, and pore diameter for the synthesized DAMP inhibitor (Fig. 2). The isotherm profile has the type II in Langmuir types. Figure 2 shows that the DAMP compound has a surface area of 9.07 m^2^/g, a pore volume of 0.13 cm^3^/g, and a pore size of 56.49 nm.

### Weight loss measurements

**Figure 3:**
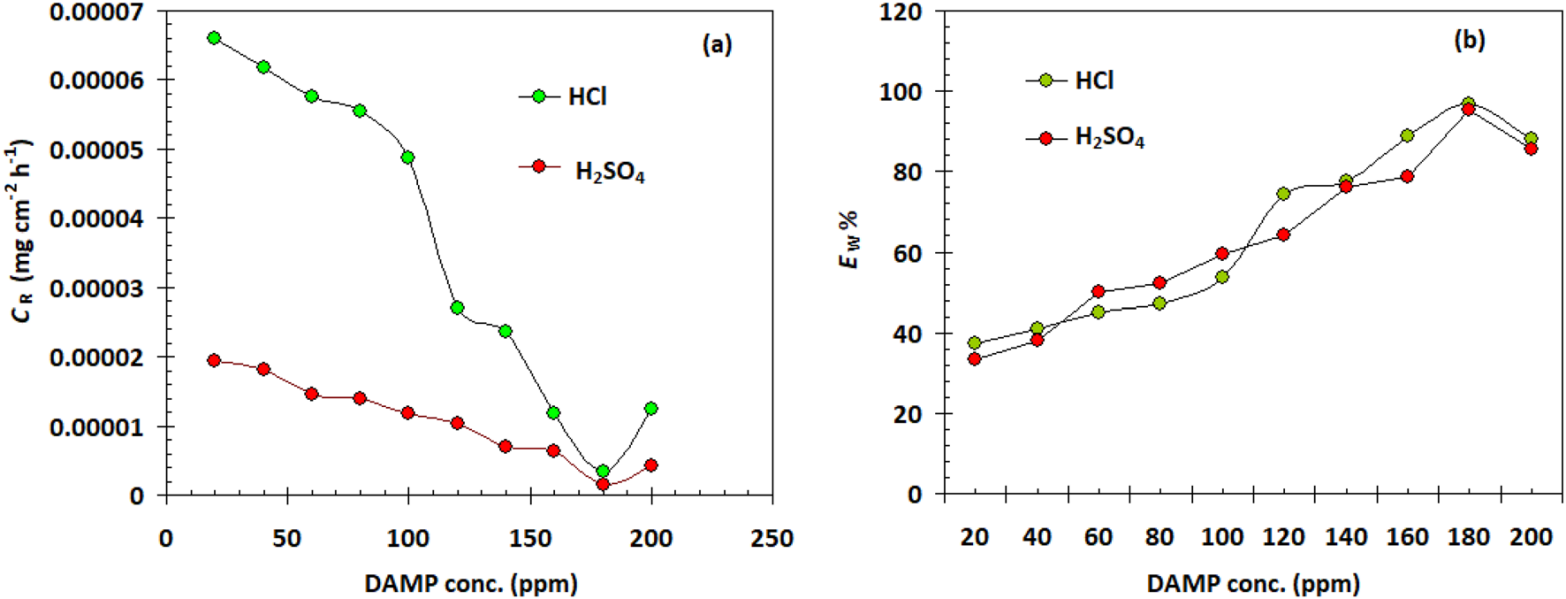
Variation of the corrosion rate (**a**) and protection efficiency (**b**) with DAMP concentration for copper in 1.0 M HCl and 1.0 M H_2_SO_4_ at 25 °C.

Copper sample corrosion rates were assessed using weight loss measurements in both acid solutions with varying concentrations of the DAMP inhibitor. It can be calculated using the fluctuation in weight per unit surface area and time. This phrase is suitable when discussing uniform and universal corrosion processes. Figure 3 depicts the variation in corrosion rate in 1 M HCl and 1 M H_2_SO_4_ in the presence and absence of different doses of the DAMP. It is evident that in every example studied at the corrosion rates decrease as the DAMP concentration increases. According to this, the DAMP reduces the rate of copper corrosion in both acids. The amount of inhibition depends on the DAMP chemical concentration and the properties of the corrosive media. The protective efficacy *E*_W_% of the DAMP in both acid solutions was calculated using Eq. (2). Table 2 presents and lists the outcomes. The *E*_W_% increases with concentration until the optimal DAMP concentration is reached at 180 ppm for HCl and H_2_SO_4_. This outcome is caused by the fact that the amount of inhibitor that is adsorbs and covers the copper surface increases as inhibitor concentration rises (see Fig. 3 and Table 2).

**Table 2:** The effect of DAMP concentration on the corrosion rate and protection efficiency for copper in 1.0 M HCl and 1.0 M H_2_SO_4_.

DAMP conc. (ppm)	*C*_R_ (mg cm^-2^ h^-1^) × 10^–5^		*E*_W_ %	
	1.0 M HCl	1.0 M H_2_SO_4_	1.0 M HCl	1.0 M H_2_SO_4_
Blank	10.50 ± 0.25	2.92 ± 0.16	–	–
20	6.59 ± 0.21	1.94 ± 0.12	37.2	33.5
40	6.18 ± 0.19	1.81 ± 0.10	41.1	38.0
60	5.76 ± 0.18	1.46 ± 0.17	45.1	50.0
80	5.55 ± 0.19	1.39 ± 0.17	47.1	52.3
100	4.86 ± 0.17	1.18 ± 0.19	53.7	59.5
120	2.71 ± 0.13	1.04 ± 0.09	74.2	64.2
140	2.36 ± 0.15	6.94 ± 0.24	77.5	76.1
160	1.18 ± 0.14	6.25 ± 0.26	88.7	78.5
180	0.347 ± 0.02	0.139 ± 0.11	96.6	95.2
200	0.417 ± 0.01	0.146 ± 0.11	96.0	95.0

**Figure 4:**
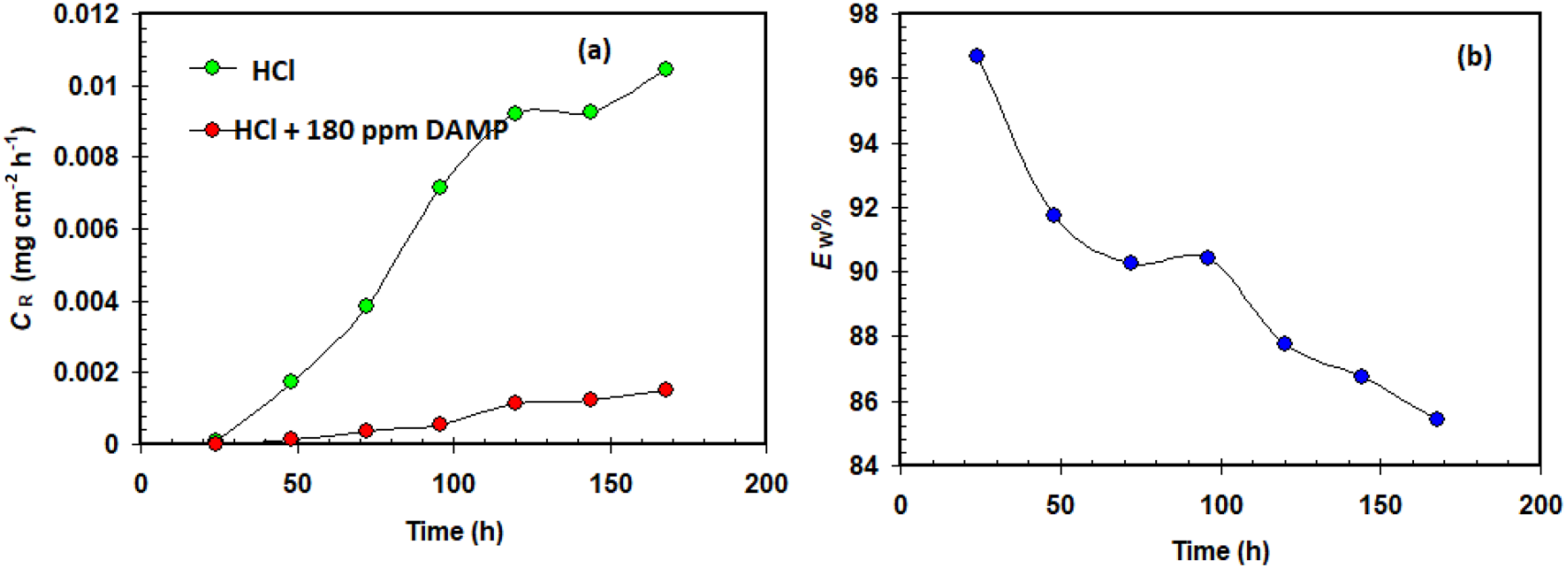
Variation of the corrosion rate (**a**) and protection efficiency (**b**) with time of immersion for copper samples in 1.0 M HCl at 25 °C.

Figure 4 represents the change of corrosion rates with respect to time (1–7 days) for copper samples in 1 M HCl acid solution and without 180 ppm of the DAMP. It is clear that over time the rate of corrosion increases in both cases, but in the absence of the DAMP, the rate of corrosion is much greater. This confirms the importance of the DAMP in reducing the severity of corrosion. It is also obvious that the *E*_W_ % decrease from 96.6% after immersion copper in 1 M HCl solution for 24 h to 85.4% after 168 h (7 days).

### Electrochemistry measurements

**Figure 5:**
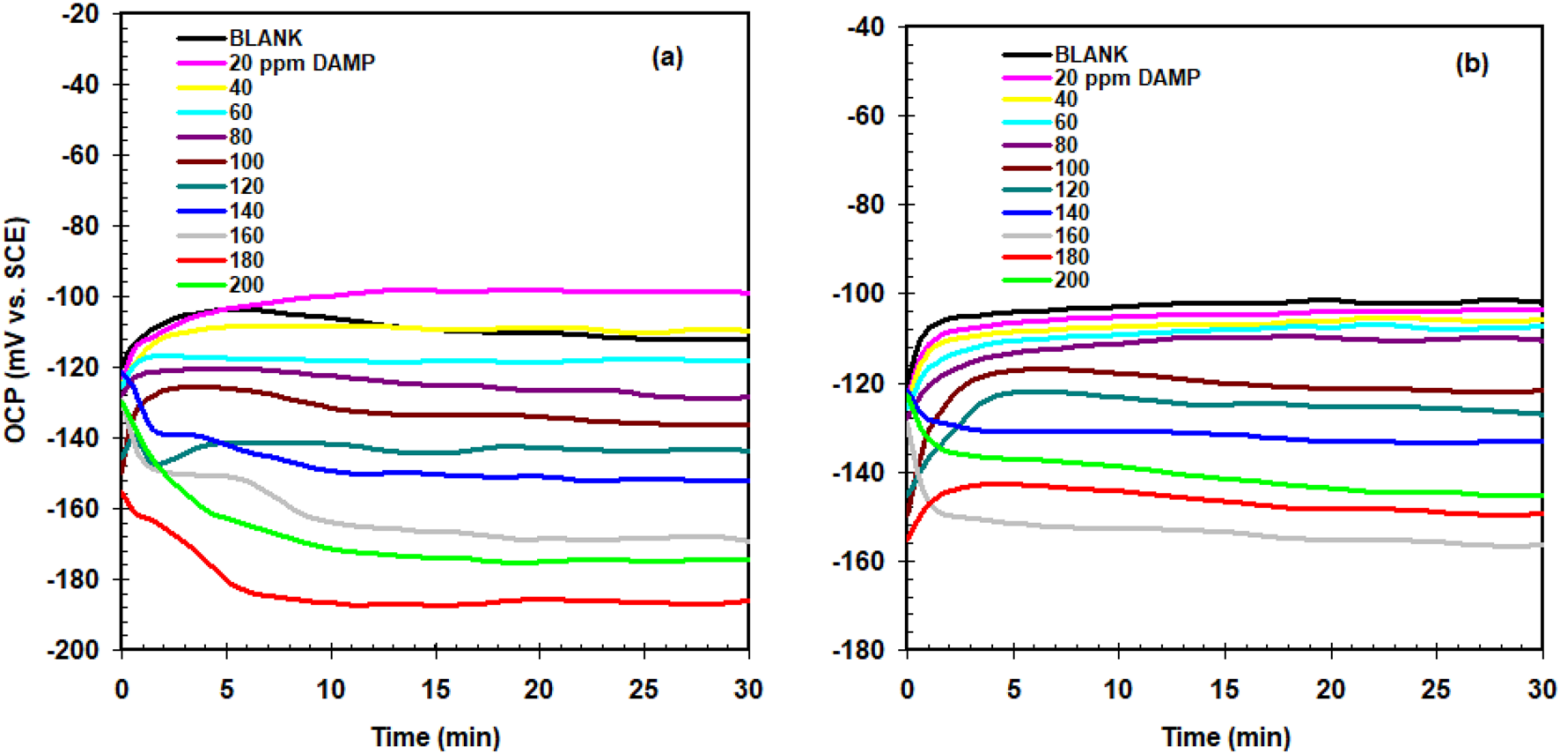
Variation of the OCP as a function of time recorded for copper in 1.0 M H_2_SO_4_ (**a**) and 1.0 M HCl (**b**) solutions without (blank) and with DAMP at 25 °C.

After 30 min of testing the open circuit potential (OCP) values for copper in acid solutions with and without DAMP, the resulting curves are displayed in Fig. 5. Figure 5a,b demonstrates that when the DAMP content increases, the open-circuit potential comes more negative, indicating that the DAMP can effectively minimize the cathode response.

**Figure 6:**
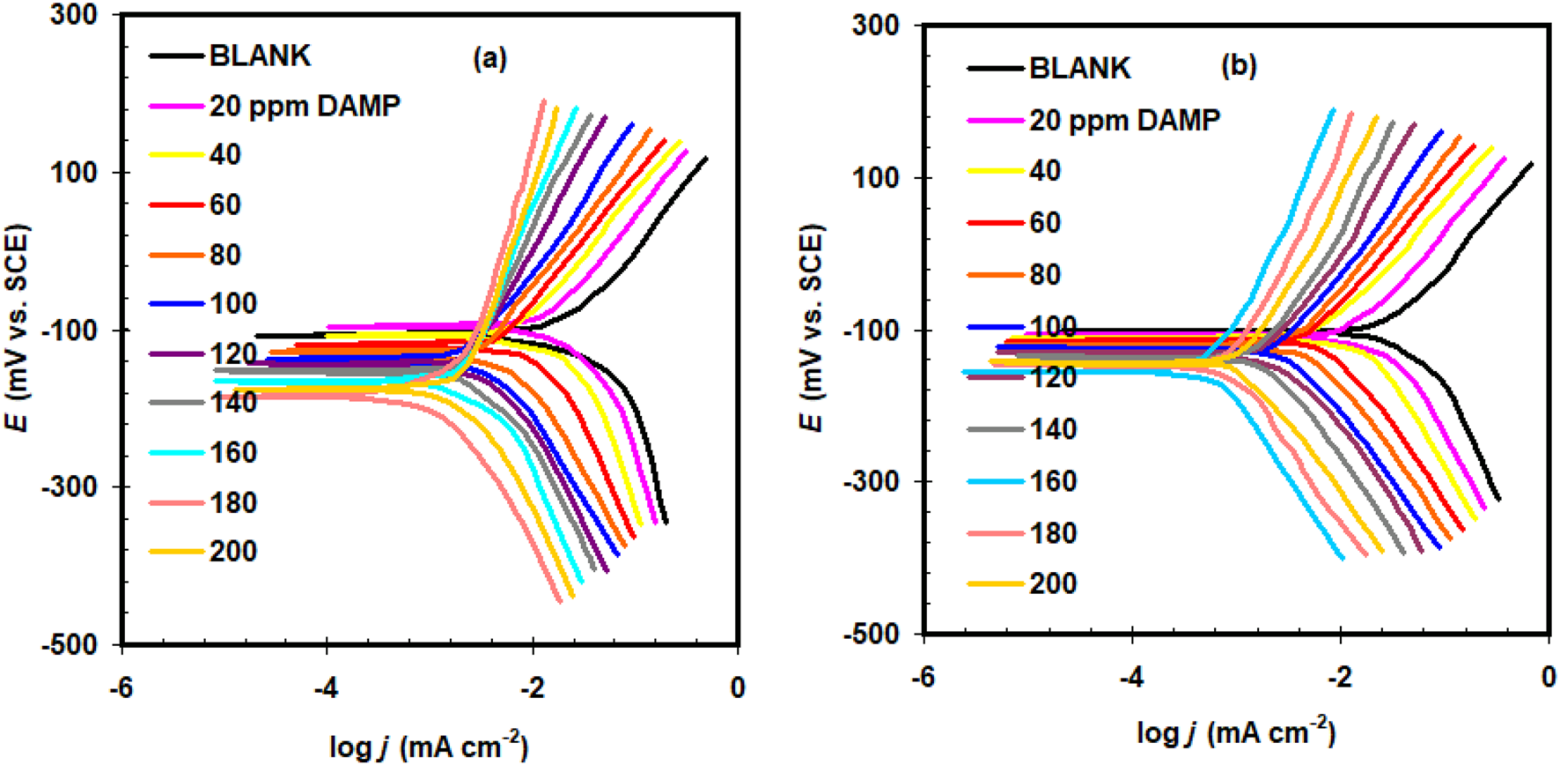
Polarization curves of copper in 1.0 M H_2_SO_4_ (**a**) and 1.0 M HCl (**b**) solutions without (blank) and with DAMP at a scan rate of 0.125 mV/s and at 25 °C.

Figure 6 depicts the Tafel curves for copper-immersed 1.0 M HCl and 1.0 M H_2_SO_4_ solutions with and without DAMP. The Tafel plots were used to estimate the polarization parameters (*i*_corr_ corrosion current density, *E*_corr_ corrosion potential, b_a_ and b_c_ Tafel slopes), and the inhibition efficiency (*E*_p_%) was computed using Eq. (3); their values are reported in Table 3^39–41^.

3 $$E_{{P}} {\% } = \frac{{i_{{{corr(0)}}} - i_{{{corr}}} }}{{i_{{{corr(0)}}} }} \times {100}$$

where *i*_corr(0)_ is recoded in blank solution.

**Table 3:** The effect of DAMP concentration on the polarization parameters for copper 1.0 M HCl and 1.0 M H_2_SO_4_.

Solution	DAMP conc. ppm	*−E*_corr_ mV (SCE)	*b*_a_ (mV dec^-1^)	*−b*_c_ (mV dec^-1^)	*i*_corr_ μA cm^-2^	*E*_p_%
1 M HCl	Blank	102 ± 2.5	98	171	25.87 ± 1.9	–
	20	106 ± 1.9	91	165	17.45 ± 1.8	32.5
	40	110 ± 2.2	98	154	15.72 ± 1.7	39.2
	60	115 ± 2.5	86	132	15.15 ± 1.2	41.4
	80	120 ± 2.6	92	130	14.07 ± 1.1	45.6
	100	123 ± 2.5	104	119	12.49 ± 0.9	51.7
	120	130 ± 2.7	102	109	7.86 ± 0.4	69.6
	140	135 ± 2.8	89	124	5.63 ± 0.5	78.2
	160	156 ± 3.1	94	165	0.969 ± 0.2	96.2
	180	147 ± 2.9	88	112	2.43 ± 0.3	90.6
	200	142 ± 3.3	87	111	2.27 ± 0.2	91.2
1 M H_2_SO_4_	Blank	109 ± 1.3	95	165	34.90 ± 1.3	-
	20	96 ± 1.4	93	156	24.91 ± 1.5	28.6
	40	108 ± 2.1	92	143	22.05 ± 1.6	36.8
	60	119 ± 2.7	83	133	18.39 ± 1.3	47.3
	80	128 ± 3.2	80	125	16.22 ± 1.1	53.5
	100	137 ± 3.5	76	126	13.43 ± 1.0	61.5
	120	142 ± 3.5	95	119	9.03 ± 1.2	74.1
	140	151 ± 3.6	92	123	7.71 ± 0.8	77.9
	160	165 ± 2.5	101	133	5.68 ± 0.7	83.7
	180	184 ± 2.7	94	143	2.58 ± 0.5	92.6
	200	175 ± 2.6	82	110	4.43 ± 0.2	87.3

In every instance, increasing the concentration of the DAMP causes the *i*_corr_ values to fall. We observed that the *E*_corr_ values change slightly with DAMP concentrations. With regard to *E*_corr_ in blank solution, the maximum shifting is less than > 85 mV. The DAMP is a mixed type inhibitor, according to this. The fact that the DAMP changes both b_a_ and b_c_ indicates that it affects the processes of both cathodic and anodic responses. Even at low DAMP doses, the corrosion inhibition is apparent. The increase in DAMP concentration is directly proportional to *E*_p_% values. The maximum *E*_p_% was obtained at 160 ppm in the case of 1.0 M HCl (i.e., 96.2%) and at 180 ppm in the case of 1.0 M H_2_SO_4_ (i.e., 92.6%) (Table 3). This data is highly compatible with the weight loss measurements. No discernible changes in inhibition % values appear to have been found, regardless of the approaches employed. It was discovered that the inhibition percentages acquired by weight loss measurements were a little bit greater than those obtained through polarization measurements. This is because while employing the weight loss approach, the copper surface is exposed to the DAMP for a longer amount of time^42,43^.

DAMP is organic compound that possess excellent anti-corrosion properties, particularly in acidic environments. The main mechanism of its anti-corrosion action in acid can be attributed to their ability to form a protective sheet on the copper surface. Here are the key mechanisms involved:

*Adsorption*: DAMP has a high affinity for copper surfaces. When introduced into an acidic environment, this compound undergoes adsorption onto the copper surface. The adsorption occurs through chemical interactions between the DAMP molecules and the copper surface, such as coordination bonds or chemical reactions at the copper interface.

Formation of a defending film: Once adsorbed on the copper surface, DAMP undergoes chemical reactions with copper ions or protons present in the acidic environment. These reactions lead to the formation of a protective film or layer on the copper surface. The film acts as a barrier between the copper and the corrosive environment, preventing direct contact and inhibiting the corrosion process.

*Passivation*: The protective film formed by DAMP acts as a passivating layer. It inhibits the electrochemical reactions that drive corrosion, such as copper dissolution and the reduction of oxygen or other corrosive species. The passivating film impedes the movement of corrosive agents towards the copper surface, reducing the rate of corrosion.

**Figure 7:**
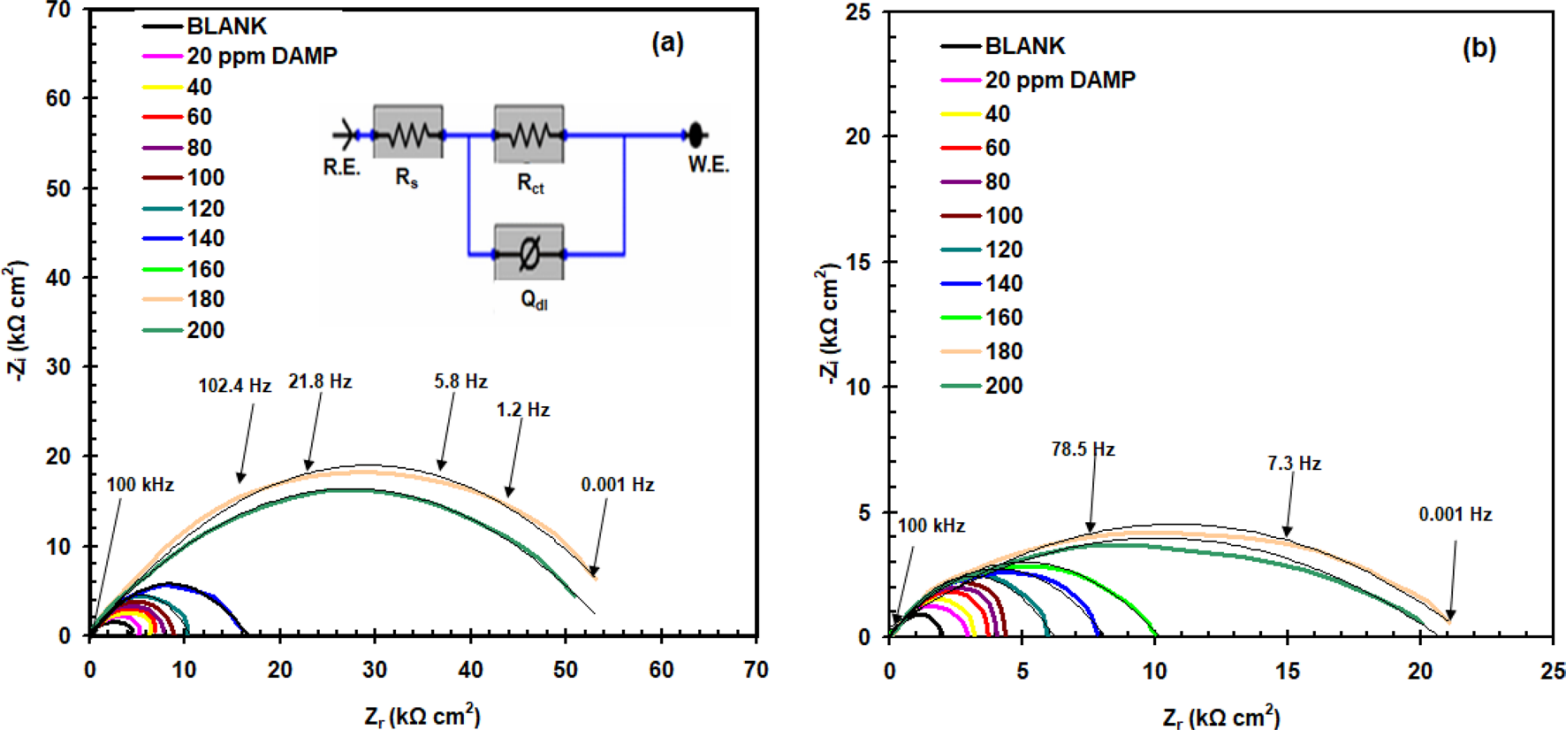
Nyquist curves of copper in 1.0 M H_2_SO_4_ (**a**) and 1.0 M HCl (**b**) solutions without (blank) and with DAMP compound at the corrosion potential and at 25 °C, the equivalent circuit insert in Fig. 7a.

Figure 7 depicts the EIS curves (Nyquist) for copper-immersed 1.0 M HCl (Fig. 7a) and 1.0 M H_2_SO_4_ (Fig. 7b) solutions with and without DAMP. The figure shows that incorporating the DAMP to 1.0 M HCl and 1.0 M H_2_SO_4_ solutions expanded the width of the Nyquist curve, indicating an increase in charge transfer resistance. The resulting graphs were analyzed and simulated using the recommended circuit, which is shown in Fig. 7a. *R*_ct_ symbolizes the charge transfer resistance, *R*_s_ symbolizes the solution resistance, and the constant phase element (*Q*_dl_) substitutes for the ideal capacitor. Table 4 presents the effect of DAMP concentration on the EIS parameters for copper 1.0 M HCl and 1.0 M H_2_SO_4_. The very low goodness of fit (χ^2^) readings in Table 4 supports the validity of the fitting approaches. It is discovered that increasing DAMP concentration causes an increase in *R*_ct_ and a decrease in *Q*_dl_, which is due to DAMP inhibitor adsorption on copper surface. “*n*” denotes the surface roughness coefficient. With *n* = 1, the *Q*_dl_ functions as a pure capacitor.

**Table 4:** The effect of DAMP concentration on the EIS parameters for copper 1.0 M HCl and 1.0 M H_2_SO_4_.

Solution	DAMP conc. ppm	*R*_ct_ kΩ cm^2^	*Q*_dl_ μF cm^-2^	χ^2^	*n*	*η*_R_%
1 M HCl	Blank	3.8 ± 0.24	435 ± 3.6	1.2 × 10^−3^	0.87	–
	20	5.1 ± 0.35	412 ± 3.2	1.3 × 10^−3^	0.88	25.4
	40	6.2 ± 0.18	387 ± 3.0	1.3 × 10^−3^	0.84	38.9
	60	6.7 ± 0.14	356 ± 4.0	1.4 × 10^−3^	0.83	43.2
	80	7.3 ± 0.23	341 ± 2.6	1.7 × 10^−3^	0.90	47.9
	100	7.9 ± 0.26	320 ± 2.6	1.0 × 10^−3^	0.91	51.8
	120	10.8 ± 0.30	276 ± 2.2	0.9 × 10^−3^	0.83	64.8
	140	16.3 ± 0.42	266 ± 3.1	1.8 × 10^−3^	0.82	76.6
	160	53.9 ± 1.12	231 ± 2.4	1.1 × 10^−3^	0.84	92.9
	180	50.2 ± 1.23	222 ± 3.7	1.2 × 10^−3^	0.88	92.4
	200	51.3 ± 0.96	187 ± 1.5	1.4 × 10^−3^	0.87	92.5
1 M H_2_SO_4_	Blank	2.2 ± 0.20	687 ± 2.5	1.1 × 10^−3^	0.92	-
	20	2.9 ± 0.12	642 ± 3.0	1.2 × 10^−3^	0.91	24.1
	40	3.2 ± 0.14	601 ± 3.2	1.5 × 10^−3^	0.91	31.2
	60	3.7 ± 0.20	543 ± 4.2	0.8 × 10^−3^	0.88	40.5
	80	4.0 ± 0.26	533 ± 2.7	0.9 × 10^−3^	0.89	45.0
	100	4.3 ± 0.19	511 ± 2.6	1.2 × 10^−3^	0.87	48.8
	120	5.9 ± 0.18	498 ± 2.8	1.3 × 10^−3^	0.86	62.7
	140	7.2 ± 0.27	432 ± 2.9	1.3 × 10^−3^	0.85	69.4
	160	9.8 ± 0.29	423 ± 2.6	1.0 × 10^−3^	0.86	77.5
	180	22.9 ± 1.2	417 ± 2.2	1.1 × 10^−3^	0.85	90.3
	200	22.8 ± 1.1	374 ± 1.4	1.1 × 10^−3^	0.83	90.3

The inhibition effectiveness (*η*_R_%) was determined employing the corresponding equation:

4 $$\eta_{{R}} \% = \frac{{R_{{{ct}}} - R_{{{cto}}} }}{{R_{{{ct}}} }} \times 100$$

where *R*_cto_ is recoded in blank solution.

EIS measurements yield similar percentage corrosion suppression efficiency as polarization and weight loss measures (Table 4).

The extracted results from studies conducted on corrosion inhibition behavior of different organic compounds and their derivatives such as azoles, amines, amino acids, and many others used for protection of copper in phosphoric and hydrochloric acids solutions are given in Table S1. As can be seen in Table S1,^44–53^ DAMP inhibitor is one of the highest corrosion inhibition efficiencies, even using small concentrations of it. It is also an easy and novel compound to prepare with a high yield and economic method through a one pot three component reaction at room temperature without the need to use heat. It dissolves easily in acidic media without the need to use other organic solvents.

### Computational studies

**Figure 8:**
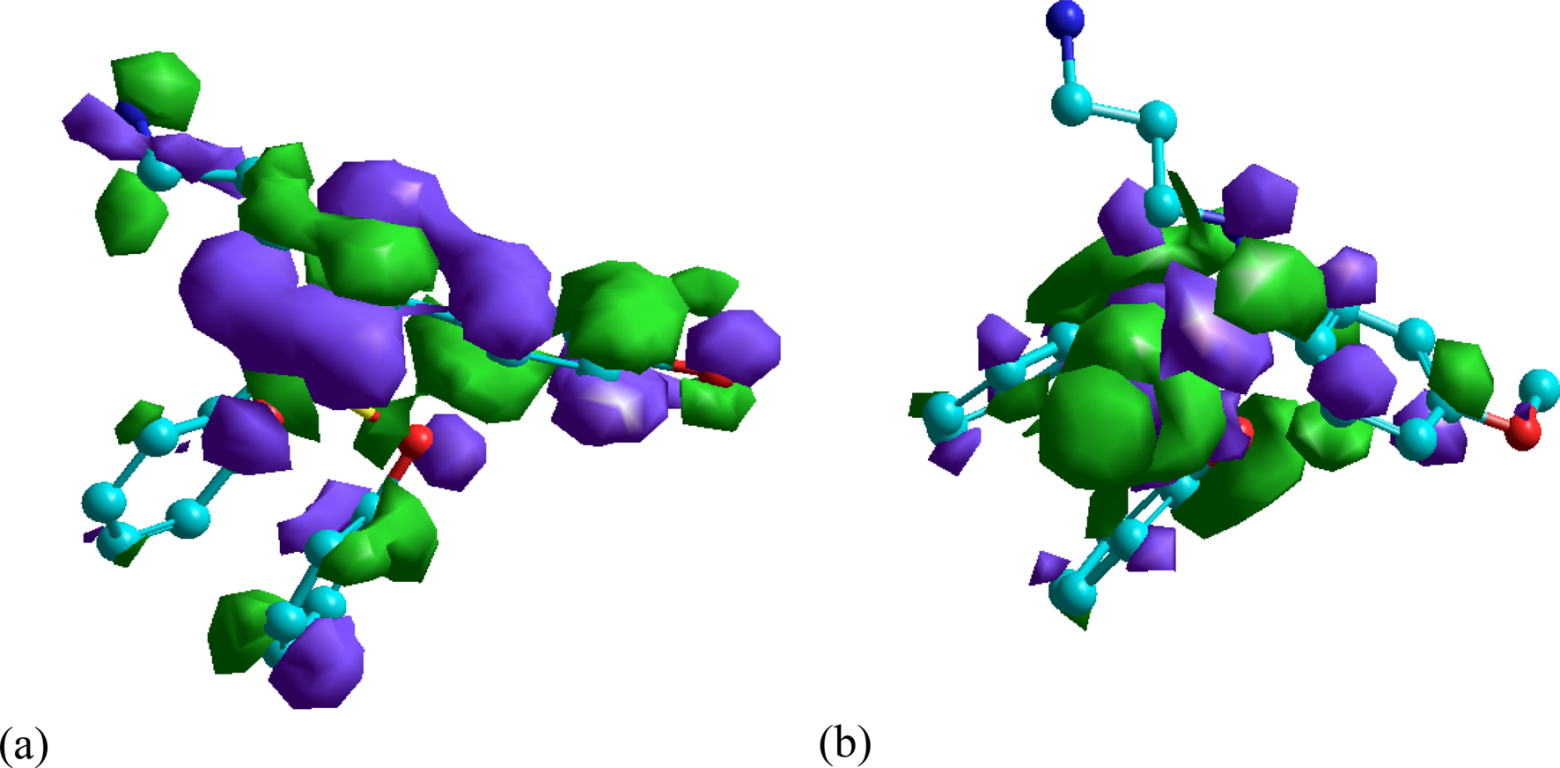
HOMO (**a**) and LUMO (**b**) distributions in DAMP molecule.

The quantum chemical parameters extracted from the output file such as E_H_ (the energy of the highest occupied molecular orbit) and E (the energy of the lowest unoccupied molecular orbit), the global hardness (η), softness (σ), and the energy of back-donnation (ΔE_b-d_) (see Table 1). Concerning Table 1, the high negative value of E_H_ and slight negative value of E_L_ declare that the investigated molecules not only have great ability for electron donation to the vacant d orbital of Cu but can also accept electron from the metal surface. In addition, the donation and back-donation of the investigated molecule identified also from the lower value of ΔE_b-d_. Moreover, Lower energy gap means easy jumping of electron HOMO to LUMO of the other molecule which may result in high efficiency values^54–57^. Figure 8 shows the HOMO and LUMO of DAMP inhibitor. According to various researches^58–60^, the displacement of water molecules allows the inhibitors to form a protective layer on the metal surface, as all inhibitors have higher dipole moments compared to water (1.8 Debyes) preventing corrosion. Additionally, the strong interaction between the high dipole inhibitors and the metal surface enhances their ability to adsorb onto the surface, further enhancing their inhibition efficiency.

The hardness and softness of the molecule is another important parameter that describe the reactivity of the examined molecule^61^. Lower value of η and higher value of σ indicating the lower deformation resistance of the e’s cloud of the atoms and hence higher reactivity of the molecule.

**Figure 9:**
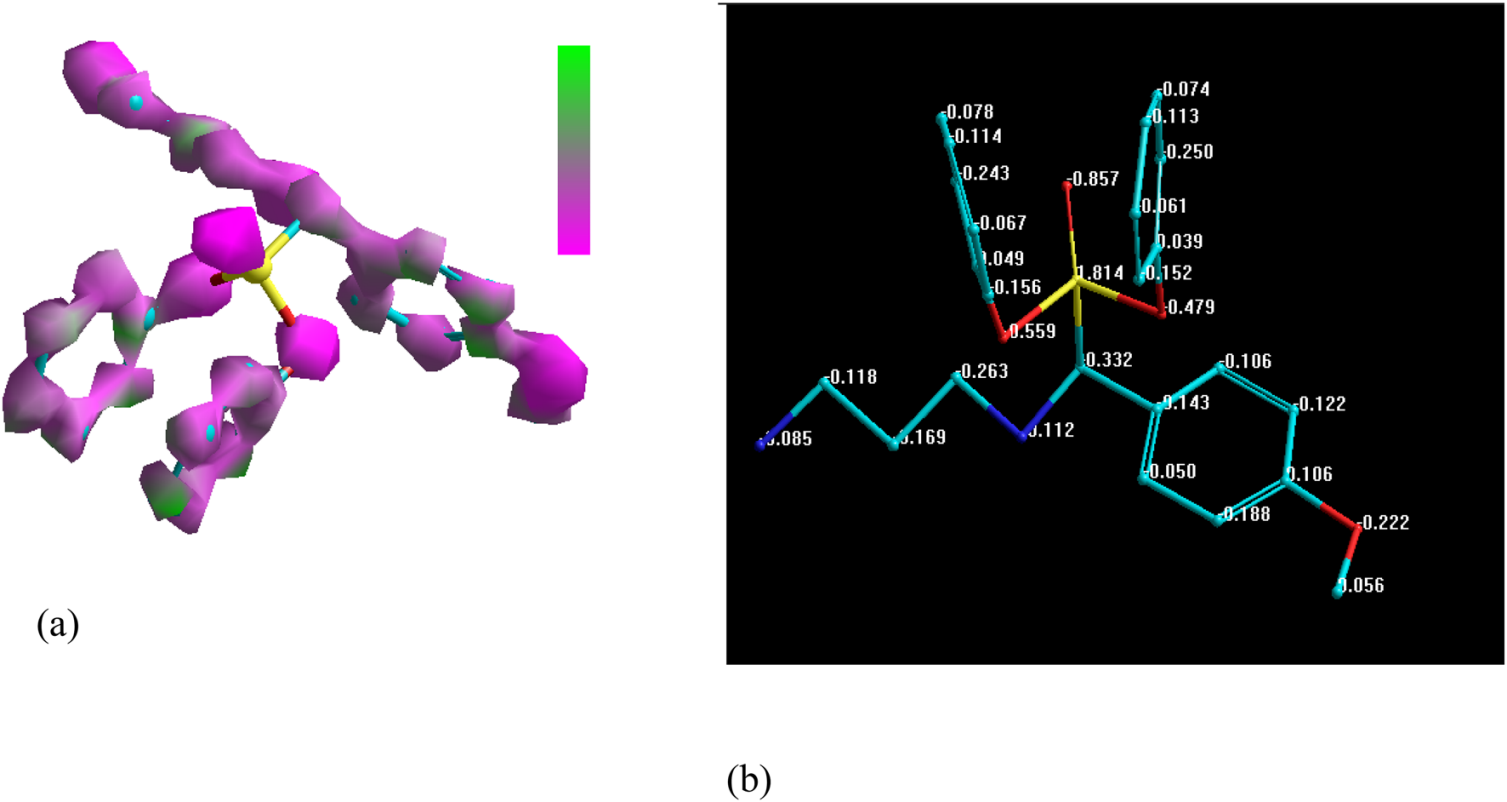
Distribution of charge density (**a**), and (**b**) Mulliken atomic charges calculated for DAMP molecule.

The electron density distribution in the inhibitors has a significant impact on how the inhibitor interacts with the surface of copper. Strong adsorption of the molecule on the metal surface would arise from regions of the molecule with high electron densities preferentially donating electrons to Cu. As clear from Fig. 9a, the electron are distributed over the most molecule which mean that the adsorption of the molecule from different active sites resulting excellent protection. Figure 9b shows the charge of each atom in the molecule; negatively charged atoms possess the great donation ability to the metal surface.

### Effect of temperature and thermodynamic study

In studying the corrosion phenomenon, temperature is generally a crucial factor. Because the reaction solution/metal is prevented by the temperature increase. As a result, it is possible to think about the type of inhibitor that binds to the metal surface and determine whether the inhibitor remains stable as the temperature rises^62^.

The impact of temperature on the efficacy of DAMP as a corrosion inhibitor is investigated using weight loss measurements on copper corrosion in the range of 298–328 k in the 1.0 M HCl and H_2_SO_4_ solutions in addition to and without the inhibitor for 24 h of immersion. The information regarding corrosion rate and inhibition efficiency is shown in Table 5. The findings show that when the temperature rises, copper corrosion rates in acid solutions both inhibited and uninhibited increase. Higher temperatures generally accelerate chemical reactions, including the corrosion process. Increasing the temperature shifted the adsorption–desorption balance towards desorption on the Cu surface^63^. The steady decline in inhibitory effectiveness as temperature rises (298, 308, 318, and 328 K) points to a physisorption process. Although the difference was not very noticeable, it does show that the inhibitor is stable at higher temperatures. Even at higher temperatures, the DAMP molecule is a potent inhibitor. Adsorption of inhibitor on metal surface occurs by two paths by either physisorption or chemisorption. Physisorption involves electrostatic force of interaction between ionic charges at the metal and the solution interface. Physisorption is relatively stable at low temperatures since the heat of adsorption is low. On the other hand, chemisorption involves charge transfer from the inhibitor molecules to the metal surface through a coordinate bond which is more stable type of adsorption than physisorption even at high temperatures so the inhibition efficiency only decreased from (96.4% to 85%) and (95.4% to 82.6%) for HCl and H_2_SO_4_ respectively at 328 K.

**Table 5:** The corrosion rate and inhibition efficiency for copper after 24 h of immersion at various temperatures in 1 M HCl and H_2_SO_4_ with and without 180 ppm of DAMP.

Temperature (K)	Solution	Corrosion rate (mg cm^-2^ h^-1^)	*E*_W_ %
298	Blank 1 M HCl	10.50 × 10^–5^	–
	180 ppm of DAMP	0.347 × 10^–5^	96.6
	Blank 1 M H_2_SO_4_	2.92 × 10^–5^	–
	180 ppm of DAMP	0.139 × 10^–5^	95.2
308	Blank 1 M HCl	32.40 × 10^–5^	–
	180 ppm of DAMP	2.70 × 10^–5^	91.6
	Blank 1 M H_2_SO_4_	45.60 × 10^–5^	–
	180 ppm of DAMP	3.88 × 10^–5^	91.4
318	Blank 1 M HCl	48.20 × 10^–5^	–
	180 ppm of DAMP	5.62 × 10^–5^	88.3
	Blank 1 M H_2_SO_4_	46.7 × 10^–5^	–
	180 ppm of DAMP	5.90 × 10^–5^	87.3
328	Blank 1 M HCl	47.90 × 10^–5^	–
	180 ppm of DAMP	7.08 × 10^–5^	85.2
	Blank 1 M H_2_SO_4_	46.90 × 10^–5^	–
	180 ppm of DAMP	7.15 × 10^–5^	87.7

It was possible to determine additional parameters, such as the activation energy (*E*_a_), the standard enthalpy (ΔH^*^), and the entropy (ΔS^*^) of the reaction, to explain the corrosion process as well as the potential mechanism of inhibitor adsorption, through analysis of the plotting of the corrosion rate as a function of temperature. The thermodynamic parameters for copper corrosion in HCl and H_2_SO_4_ solutions in the absence and presence of DAMP were calculated using the Arrhenius and Eyring-Polanyi calculations^64,65^.

5 $$C_{R} = Ae^{{\frac{{ - E_{{a}} }}{RT}}}$$

6 $$C_{R} = \frac{RT}{{Nh}}e^{{\frac{{\Delta S^{ * }_{{}} }}{R}}} e^{{\frac{{ - \Delta H^{ * } }}{RT}}}$$

where *E*_a_ apparent activation energy, *λ* the pre-exponential factor, ΔH^*^ the apparent enthalpy of activation, ΔS^*^ the apparent entropy of activation, *h* the Planck’s constant, *N* the Avogadro number, *R* the universal gas constant and *T* the thermodynamic temperature.

**Figure 10:**
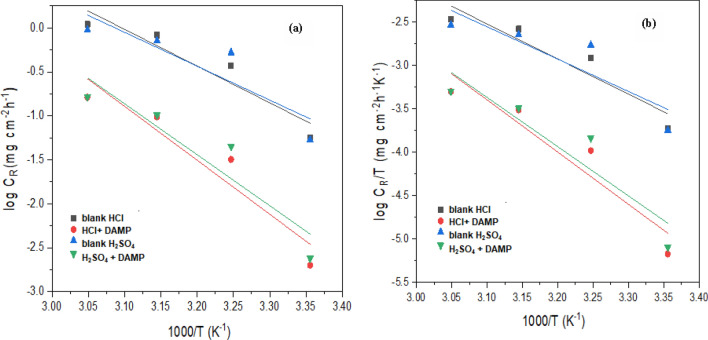
(**a**) Arrhenius and (**b**) Eyring–Polanyi plots for copper corrosion in 1 M HCl and H_2_SO_4_ in the absence and presence of DAMP.

A straight line with a slope of *E*_a_/2.303*R* was produced by plotting the log *C*_R_ vs. 1/*T*. This result is presented in (Fig. 10a). Table 6 provides the activation energy values. The information demonstrates that the activation energy *E*_a_ of copper corrosion in HCl and H_2_SO_4_ solutions with DAMP is greater than that of the control solution. Increased DAMP molecule adsorption on the metal surface could explain the rise in the apparent activation energy for copper corrosion in inhibited acid solutions^66^.

**Table 6:** The activation thermodynamic parameters in the presence and absence of DAMP.

Solution	*E*_a_ (KJ/mol)	ΔH* (KJ/mol)	ΔS* J mol^-1^ K^-1^
Blank (HCl)	23.84	23.07	−5.95
DAMP + HCl	35.06	34.23	93.42
Blank (H_2_SO_4_)	21.98	21.21	−25.9
DAMP + H_2_SO_4_	33.09	32.31	74.01

Plotting log (*C*_R_/*T*) vs. 1/*T* resulted in a straight line with (H^*^/2.303*R*) for the slope and (log *R/Nh* + S^*^/2.303*R*) for the intercept, as shown in (Fig. 10b). Table 6 lists the values for H^*^ and S^*^ that were determined from the slope and the intercept. The process of dissolving copper is endothermic, as indicated by the positive sign of enthalpies (H^*^). Additionally, the values for H^*^ are higher in the presence of the inhibitor than they are in the uninhibited solution. This implies that the energy required for copper to dissolve in acidic solutions increases when a DAMP inhibitor is present. This indicates that it is challenging to dissolve copper in acid solutions when DAMP is present. Adding DAMP inhibitor results in a large positive entropy shift (ΔS*), indicating a transition to an ordered system^67^. This implies that transitioning from reactants to activated complex results in a decrease in disordering because the activated complex indicates association rather than dissociation in the rate-determining stage^68^.

### Adsorption isotherm

By fitting the existing data, several mathematical correlations for the adsorption isotherms have been developed. The general Langmuir isotherm is shown in Eq. s7^,70^:

7 $$\frac{{C_{{{inh}}} }}{\theta } = \frac{1}{{K_{\begin{subarray}{l} {ads} \\ \end{subarray} } }} + C_{{{inh}}}$$

Where *C*_inh_ is the DAMP concentration, *K*_ads_ is the equilibrium constant of the adsorption reaction, and *θ* is the extent of metal surface coverage. Weight loss data were used to calculate the amount of metal surface covered using the relationship shown below:

8 $$\theta = \frac{{C_{{{R0}}} - C_{{R}} }}{{C_{{{R0}}} }}$$

**Figure 11:**
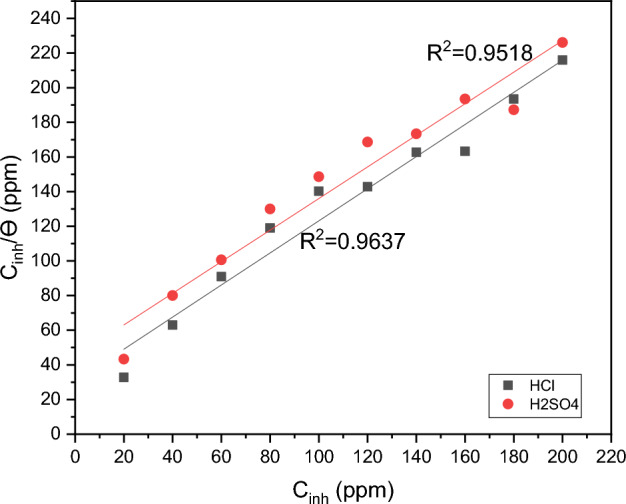
Langmuir adsorption plots for DAMP on the surface of copper in 1.0 M HCl and 1.0 M H_2_SO_4_ solutions based on data from weight loss.

According to Fig. 11, the best fit was found by the Langmuir isotherm. Regression coefficients (R^2^) practically equal to unity and the ability to draw straight lines from the graphic demonstrate how well the data fit the Langmuir adsorption isotherm. Other isotherm models with their linear regression (R^2^) are shown in Table S2.

The values of *K*_ads_ derived using the Langmuir adsorption isotherm for HCl and H_2_SO_4_ solutions (13,184 M^−1^) and (9064 M^−1^), respectively. A high *K*_ads_ value indicates higher DAMP indicator adsorption on the surface of copper.

To determine whether an adsorption process is spontaneous or not, thermodynamic considerations are required. The primary criterion for spontaneity is the standard Gibbs energy of adsorption (Δ*G*_ads_). The relationship between Δ*G*_ads_ and the equilibrium constant of adsorption is widely recognized (see Eq. 9)^71^.

9 $$\Delta G_{{{\text{ads}}}} = \, - RT{\text{ln }}\left( {{55}.{5}K_{{{\text{ads}}}} } \right)$$

where *T*: the absolute temperature, *R*: the universal gas constant (8.314 J K^−1^ mol^−1^). For solutions of HCl and H_2_SO_4_, the computed average Δ*G*_ads_ values are -33.4 and -32.5 kJ mol^−1^, respectively. This implies the existence of both physisorption and chemisorption mechanisms. The negative values of Δ*G*_ads_ for the DAMP inhibitor's adsorption on copper reflect the stability and spontaneity of adsorption processes^72^.

### Surface morphological study

#### FT-IR analysis

**Figure 12:**
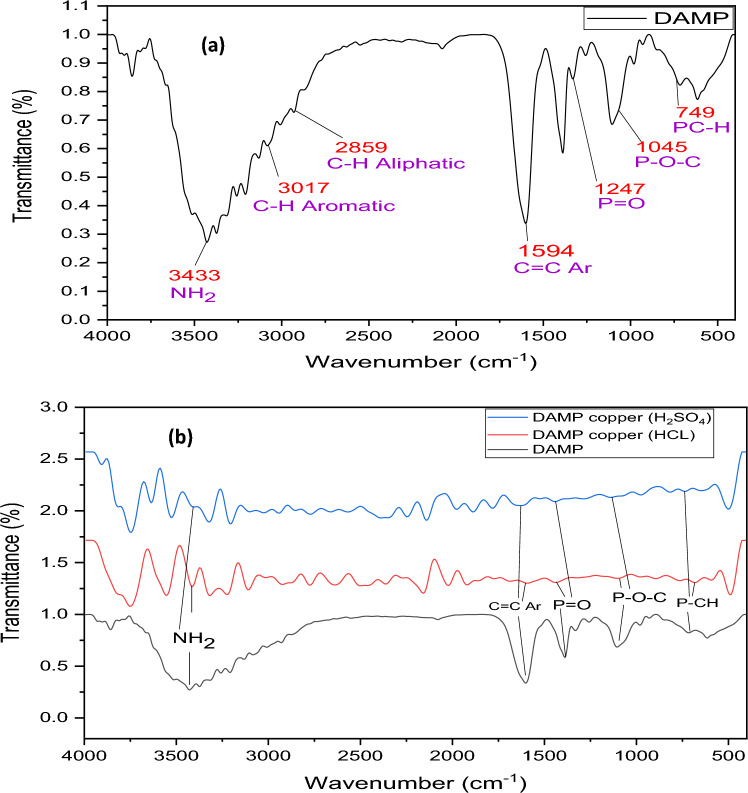
FT-IR analysis of DAMP compound (**a**), and copper surface after polarization test in the corrosive solutions (1.0 M HCl, H_2_SO_4_) containing 200 ppm DAMP.

The copper surface was subjected to FT-IR analysis following polarization in the corrosive environment (1.0 M HCl, H_2_SO_4_) containing 200 ppm DAMP in order to further demonstrate that DAMP protects the copper surface via an adsorption mechanism. Figure 12 displays the acquired spectrum in comparison to the pure DAMP. Important pure DAMP peaks may be detected in Fig. 12a thanks to the broad band that corresponds to (NH_2_) at 3441 cm^−1^. the C–H aromatic peak at 3017 cm^−1^. The C–H Alkane gas is shown by the peak at 2859 cm^−1^. The stretching of the aromatic ring (C = C–Ar) was related to the peak at 1594 cm^−1^. In addition, P = O, P–O–C, and P–CH, respectively, are responsible for the three peaks at 1247 cm^−1^, 1045 cm^−1^, and 759 cm^−1^. After the corrosion in the presence of the inhibitor, these peaks are likewise seen, albeit less intensely Fig. 12b. The AMDP compound was eventually confirmed to have been adsorbed using the N, O, and P heteroatoms and the pi electron in the C = C group of the aromatic ring by the observed peaks.

### SEM observation and EDX analysis

**Figure 13:**
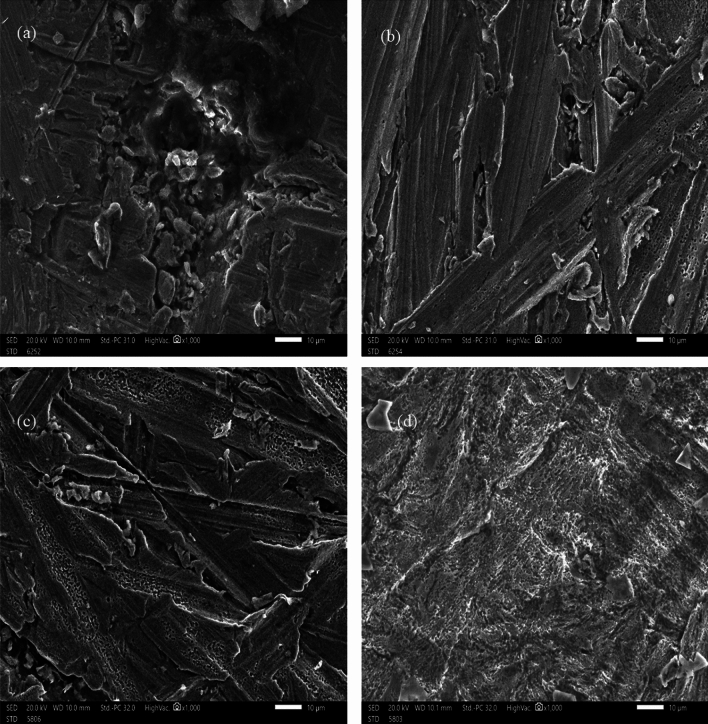
SEM images for copper surface in: (**a**) 1.0 M HCl, (**b**) 1.0 M HCl + 180 ppm of DAMP, (**c**) 1.0 M H_2_SO_4_, (**d**) 1.0 H_2_SO_4_ + 180 ppm of DAMP, after 24 h of immersion at 298 K.

The morphological characteristics of metal surface are frequently studied using SEM. Utilising SEM, the surface morphology of a copper sample immersed in 1.0 M HCl and 1.0 M H_2_SO for 24 h was examined when 180 ppm of DAMP was present. The outcomes of the experiment are shown in Fig. 13. Without DAMP, the copper specimen in the corrosive solution is severely corroded by the acidic solutions (Fig. 13a,c), resulting in the appearance of crystalline aggregates of the by-products of corrosion on the surface as well as a porous and rough outer layer. As a result of the metal's dissolution, corrosion products produced by the attack of chloride and sulfate ions severely harm the surface^73,74^. In contrast, there is significantly less damage to the copper surface when the DAMP is present (Fig. 13b,d), which supports the inhibition activity. Therefore, it may be said that the DAMP has strong copper corrosion preventing capacity. The application of a protective coating to a metal surface to stop corrosion products from forming can be used to explain these outcomes. This results from the active molecules in the DAMP adhering to surfaces.

**Figure 14:**
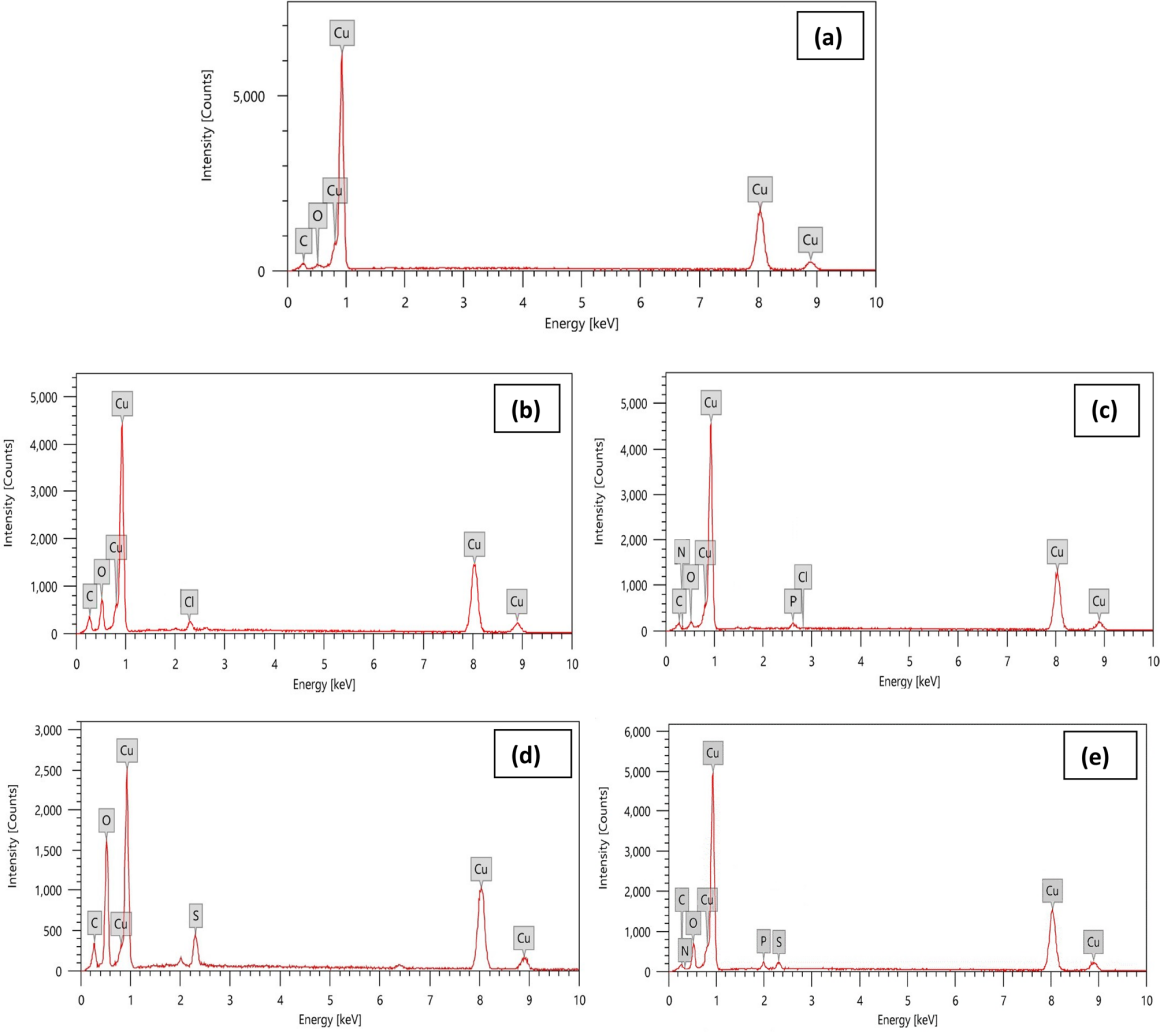
EDX analysis for copper surface: (**a**) untreated polished copper (**b**) 1.0 M HCl, (**c**) 1.0 M HCl + 180 ppm of DAMP, (**d**) 1.0 M H_2_SO_4_, (**e**) 1.0 H_2_SO_4_ + 180 ppm of DAMP, after 24 h of immersion at 298 K.

The elements on the copper surface after 24 h of immersion in 1 M HCl, H_2_SO_4_ without and with 180 ppm DAMP were identified using EDX spectra, as shown in Fig. 14, in order to learn more about the surface composition of the copper sample with and without the DAMP in 1.0 M HCl and 1 M H_2_SO_4_ solutions. Table 7 lists the mass percentages of the various elements that make up the sample both with and without the DAMP.

**Table 7:** Mass percentage contents of elements on the copper samples before and after the addition of DAMP compound in 1.0 M HCl and 1.0 M H_2_SO_4_ from EDX analysis.

Sample	Composition					
	Cu	O	Cl	S	P	N
Pure Cu (Reference)	99.01	0.71	–	–	–	–
1.0 M HCl (Blank)	72.58	11.07	1.8	–	–	–
1.0 M HCl + DAMP	87.59	3.01	0.41	–	1.8	0.43
1.0 M H_2_SO_4_ (Blank)	54.34	27.18	–	3.76	–	–
1.0 H_2_SO_4_ + DAMP	82.51	10.12	–	1.34	0.73	0.41

Prior to being exposed to the acid solutions, the pure surface of a copper sample was shown in Fig. 14a, and a mass composition of 99.01% for copper was obtained. Remaining components like O and C with ratios of 0.71% and 0.22%, respectively, were seen as a result of the surface pollution. With no DAMP inhibitor present, the copper EDS spectrum shown in Fig. 14b,d and Table 7 demonstrates how the copper peak's intensity decreased in HCl and H_2_SO_4_ (72.58, 54.34% Cu, respectively), how a characteristic chlorine peak appeared (1.8% Cl), how a characteristic sulfur peak appeared (3.76%S), and how the oxygen peak's intensity increased in HCl and H_2_SO_4_ mediums compared to virgin copper. The latter verifies that the crystalline aggregates, a corrosion product, formed on the metal surface.

The intensity peaks for Cl, S, and O are less prominent in the spectrum when DAMP inhibitor is present (Fig. 14c,e). The proportion of copper is larger than when copper is attacked without an inhibitor, as seen in Table 7. Additionally, the presence of phosphorus, nitrogen and oxygen signals on the metal surface that is inhibited suggests that the active chemical compounds that make up the DAMP compound have been adsorbed on the Cu surface by a synergistic action, which explains why the inhibition efficiency has increased (E_a_% = 96%). This demonstrates that the DAMP inhibitor's inclusion lessens copper corrosion given the presence of aromatic rings, phosphorus, and amino groups, which make it easier for the tested molecule to bind to the surface and provide good corrosion resistance, these results are not surprising. The results of the outermost characterization analysis are consistent with the electrochemical and weight loss test results. The FT-IR, SEM analysis, and experimental results are all in good accord with the EDX analysis.

### Inhibition mechanism of DAMP

The inhibition performance of an inhibitor is related to its adsorption ability on the interface of the metal. This adsorptive interaction leads to the formation of a protective layer onto the metal surface that protects the metal from corrosion^75–78^.

**Figure 15:**
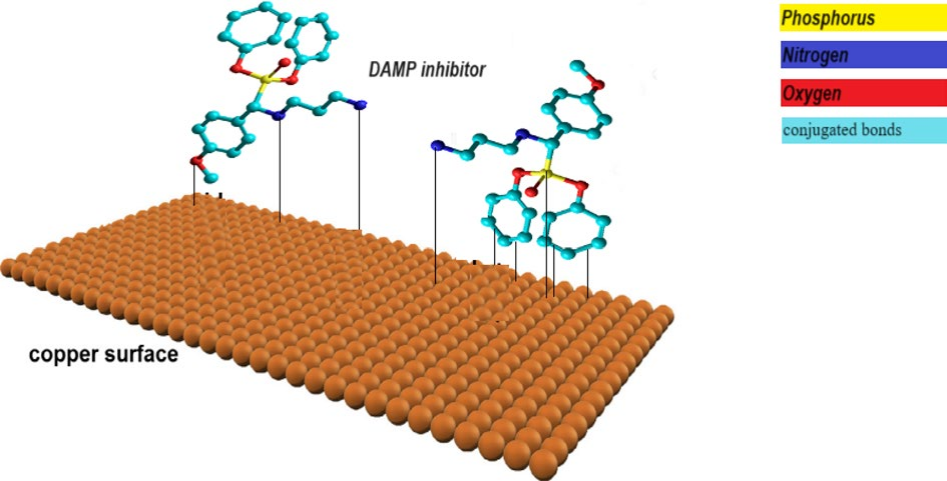
Schematic illustration of adsorption process of DAMP on copper surface.

According to Fig. 15 the adsorption process of DAMP on metal’s surface (with positive charge) may occur by the presence of heteroatoms such as nitrogen, oxygen, and phosphorous in the DAMP compound. These heteroatoms improve its adsorption process on a metal surface. The adsorption of the DAMP molecules forms a protective layer that inhibits or slows down the corrosion process. The presence of conjugated bonds and π-electrons in DAMP can influence its adsorption behavior and effectiveness. The aromatic rings and conjugated systems provide additional electron density and delocalization, which can enhance the adsorption strength and stability of the DAMP on the copper surface.

## Conclusion

1. The current work proposed the use of organophosphorus derivative (DAMP) as a new copper corrosion inhibitor for both sulfuric and hydrochloric acid solutions.
2. FT-IR, ^1^H NMR, ^31^P NMR, ^13^C NMR, and BET studies were used to characterize the molecular structure of DAMP
3. It has been discovered that the DAMP inhibitor material significantly reduces copper corrosion in both HCl and H_2_SO_4_ acids.
4. At 180 ppm, the highest levels of inhibition (96.2% for HCl and 92.6% for H_2_SO_4_) were produced.
5. SEM and EDX results revealed that DAMP compound inhibitory layers had been deposited on the copper slides surfaces, which contributed to their outstanding anticorrosion performance.
6. The adsorption of the DAMP inhibitor is categorized in great depth using the Langmuir adsorption isotherm.
7. The ability of DAMP inhibitor molecules to adsorb on copper's surface through their hetero-atoms (O, N, and P) is the main factor influencing how quickly copper specimens corrode in acidic solutions.
8. The recently developed DAMP inhibitor, which is a cheap and effective corrosion barrier, may open up a contemporary research path for developing cutting edge ecological corrosion inhibition technologies.

### Supplementary Information

Supplementary Information.

## Data Availability

The datasets used and/or analysed during the current study available from the corresponding author on reasonable request.
