# Supplementary material for: Chemical, electrochemical, and quantum investigation into the use of an organophosphorus derivative to inhibit copper corrosion in acidic environments
Source: Sci Rep. 2024 May 18;14:11395. doi: 10.1038/s41598-024-60614-5 (PMC11102478; doi:10.1038/s41598-024-60614-5)
Supplement: Supplementary file 1 — Supplementary Information. [file 41598_2024_60614_MOESM1_ESM.pdf]

Supplementary information

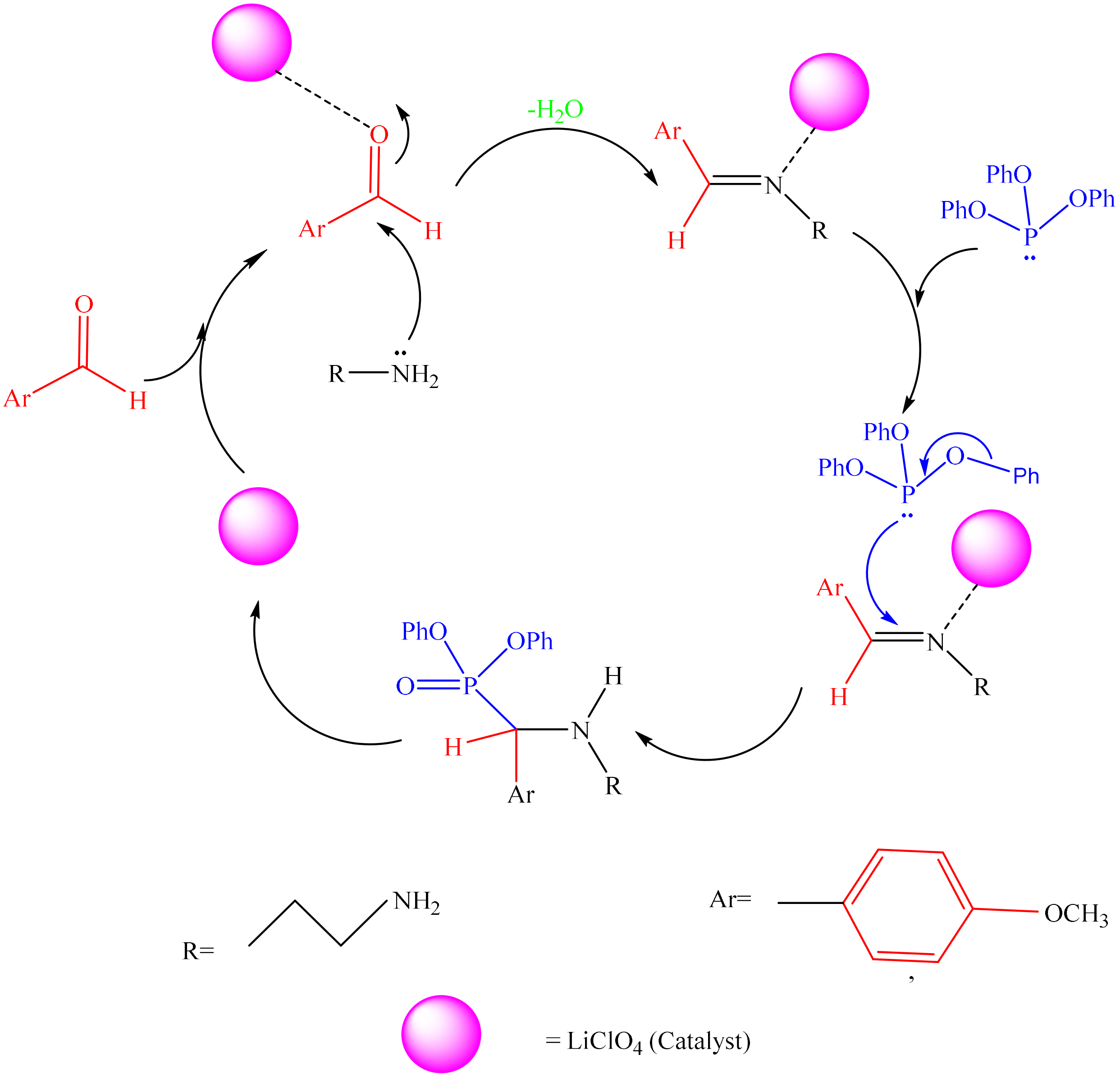

Scheme S1: Schematic reaction pathway for grafting of phenylphosphite on aniline-based sorbent.

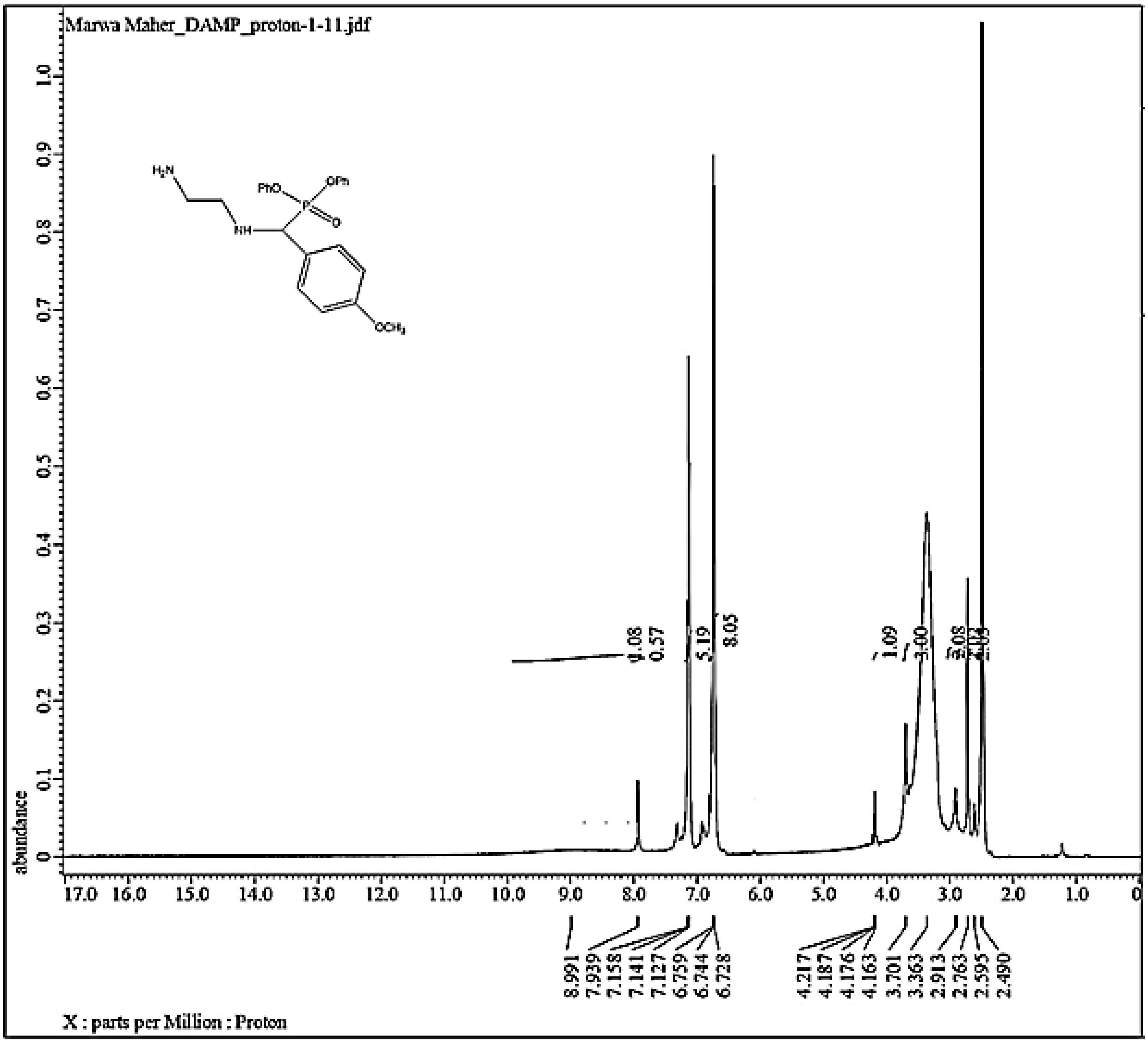

Fig. S1 ^{1}HNMR of DAMP inhibitor

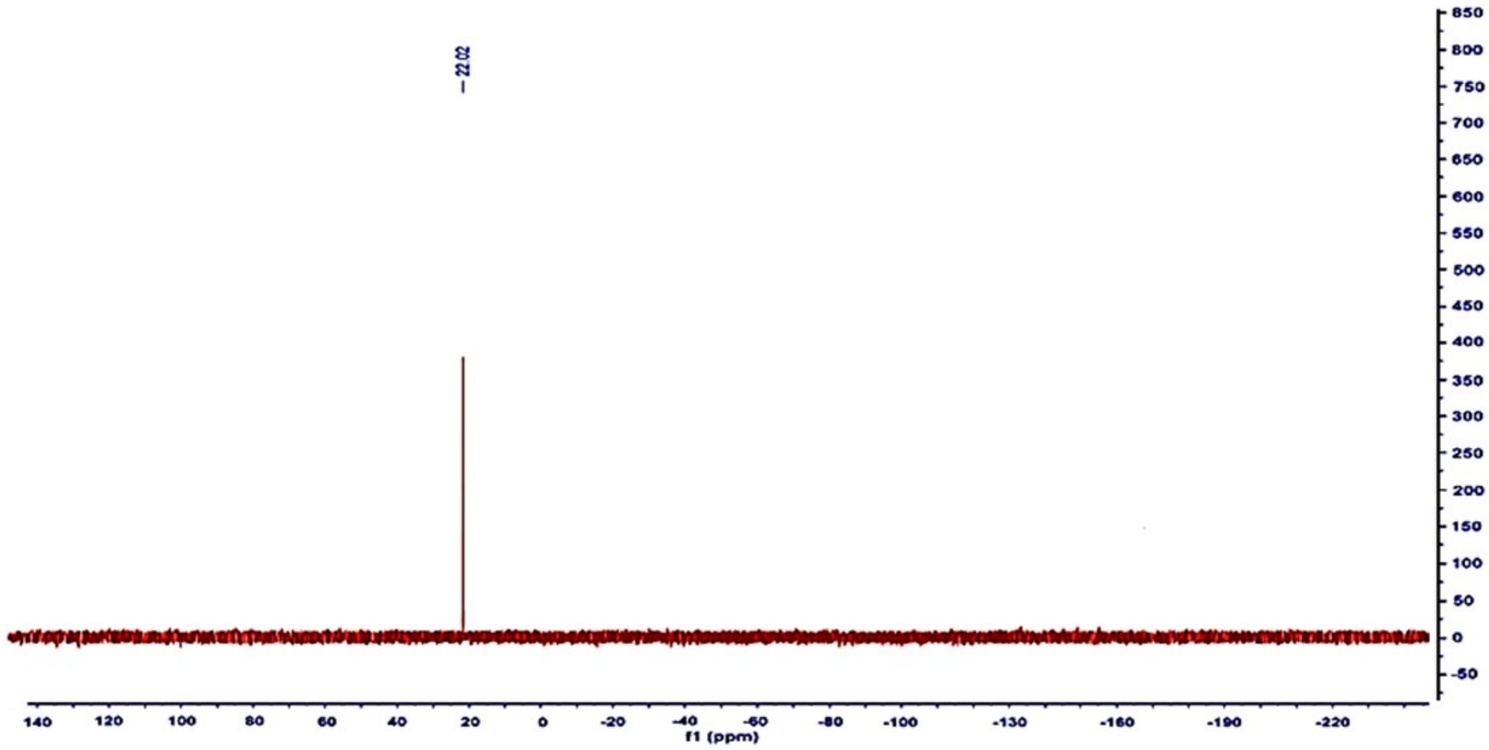

Fig. S2 ^{31}PNMR of DAMP inhibitor

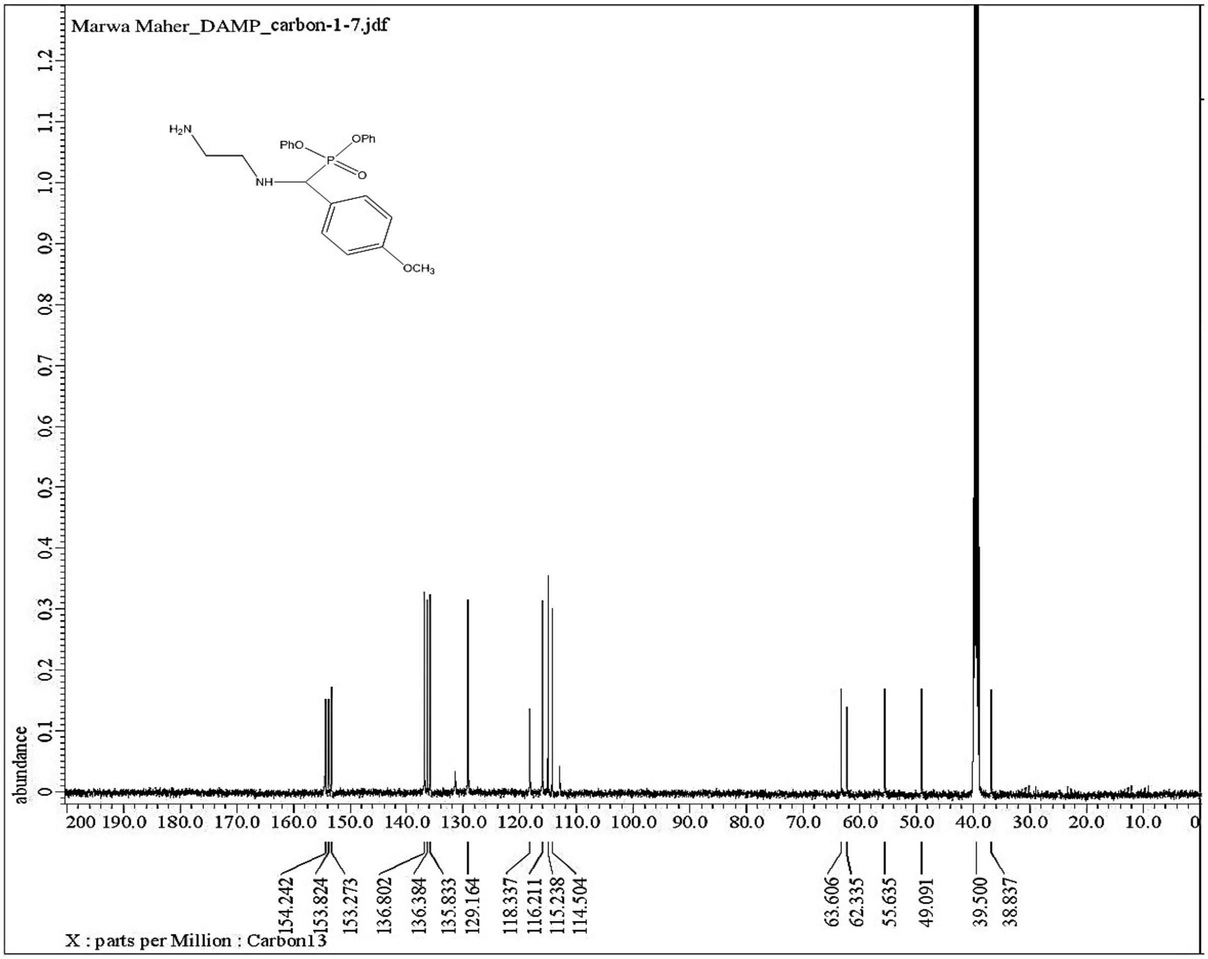

Fig.S3 ^{13}CNMR of DAMP inhibitor

Table S1: The results of the comparative study of the efficiency of copper corrosion inhibition in sulfuric and hydrochloric acids

Inhibitor group	inhibitor	Concentration	Solution	Efficiency%	Ref.
Azole	Benzotriazole (BTAH)	5 mM	1.0 M H2SO4	87 pp, 90 EIS	[44]
	Nitrotetrazolium blue chloride (NTBC)	1.22 mM	0.1 M H_2SO_4	78.55 PP, 90.24 EIS	[45]
	5-Methyl-2,4-dihydropyrazol-3-one (MHPO)	1 mM	0.1 M H_2SO_4	88.8 EIS, 76.47 Wl	[46]
	4-Amino-4H-1,2,4-triazole-3thiol (ATT)	2.58 mM	0.5 M HCl	96.09%	[47]
Amino acid	Threonine	1 mM	0.5 M HCl	83.40 pp, 87.70 EIS	[48]
Thiol	benzenethiol (BT)	1 mM	1.0 M H_2SO_4	50.6 wl	[49]
Pharmaceutical drug	Ciprofloxacin	1 mM	0.5 M H_2SO_4	88 pp, 96 EIS	[50]
Carboxylic acids	Indole-3-carboxylic acid (ICA)	2 mM	0.5 M H_2SO_4	81 pp, 89 EIS	[51]
Others	2, 4-Dinitrophenylhydrazine	0.1 mM	1.0 N H_2SO_4	85% Wl	[52]
Others	pyridone derivatives	0.18 mM		91.9%	[53]
α-aminophosphonate	DAMP	0.43 mM	1 M HCl	96.7 Wl, 96.2 PP, 92.4 EIS	here
			1M H_2SO_4	95.2 Wl, 92.6 PP, 90.3 EIS	here

Table S2: Adsorption isotherm models with their linear regression (R^2)

isotherm model	R^2 HCl solution	R^2 H_2SO_4 solution
Langmuir isotherm	0.9637	0.9518
Freundlich model	0.877	0.902
Temkin model	0.912	0.934
